# Sarcopenia of kidney transplant recipients as a predictive marker for reduced graft function and graft survival after kidney transplantation

**DOI:** 10.1007/s00423-023-02836-1

**Published:** 2023-02-24

**Authors:** H. Karakizlis, N. Trudel, A. Brose, A. Reinisch, M. Reichert, A. Hecker, F. Bender, I. Askevold, L. Rainer, R. Weimer, G. A. Krombach, W. Padberg, J. Liese

**Affiliations:** 1 Department of Internal Medicine II, Division of Nephrology and Renal Transplantation, Justus-Liebig-University of Giessen, Giessen, Germany; 2 Department of General, Visceral and Thoracic Surgery, Justus-Liebig-University of Giessen, Rudolf-Buchheim-Str. 7, Giessen, Germany; 3 Department of Diagnostic and Interventional Radiology, Marienhospital Stuttgart, Stuttgart, Germany; 4 Department of Radiology, Justus-Liebig-University of Giessen, Giessen, Germany; 5 Department of General, Visceral and Oncologic Surgery, Hospital and Clinics Wetzlar, Teaching Hospital of the Justus-Liebig-University Giessen, Wetzlar, Germany

**Keywords:** Sarcopenia, HUAC, Kidney transplantation, Kidney function, Graft survival

## Abstract

**Purpose:**

The association between sarcopenia of kidney transplant recipients and outcome after kidney transplantation (KT) has not yet been fully understood and is still considered controversial. The aim of our study was to analyze the impact of pre-transplant sarcopenia on graft function, postoperative complication rates, and survival of the patients after renal transplantation.

**Methods:**

In this retrospective single-center study, all patients who underwent KT (01/2013–12/2017) were included. Demographic data, rejection rates, delayed graft function, and graft and patient survival rates were analyzed. Sarcopenia was measured in computed tomography images by the sex-adjusted Hounsfield unit average calculation (HUAC).

**Results:**

During the study period, 111 single KTs (38 women and 73 men) were performed. Living donor kidney transplants were performed in 48.6%. In total, 32.4% patients had sarcopenia. Sarcopenic patients were significantly older (59.6 years vs. 49.8 years; *p* < 0.001), had a higher body mass index (BMI = 27.6 kg/m^2^ vs. 25.0 kg/m^2^; *p* = 0.002), and were more likely to receive deceased donor kidneys (72.2% vs. 41.3%; *p* = 0.002). Interestingly, 3 years after KT, the creatinine serum levels were significantly higher (2.0 mg/dl vs. 1.5 mg/dl; *p* = 0.001), whereas eGFR (39.9 ml/min vs. 53.4 ml/min; *p* = 0.001) and graft survival were significantly lower (*p* = 0.004) in sarcopenic transplant recipients. Sarcopenic patients stayed in hospital significantly longer postoperatively than those who were non-sarcopenic.

**Conclusions:**

At the time of kidney transplantation, sarcopenia was found to predict reduced long-term graft function and diminished graft survival after KT. The early identification of sarcopenic patients can not only enable an optimized selection of recipients, but also the initiation of pre-habilitation programs during the waiting period.

**Supplementary Information:**

The online version contains supplementary material available at 10.1007/s00423-023-02836-1.

## Introduction

Kidney transplantation (KT) improves not only quality of life [1–3] but also life expectancy [4, 5] in patients with end-stage renal disease (ESRD) in comparison to patients on dialysis. Every year, more and more elderly become eligible for KT [6]. The significant discrepancy between the number of organ donors and the need for organs underlines the necessity for careful selection of transplant candidates.

As early as 2014, Ponticelli et al. stated that there are no clear guidelines for the selection of older transplant recipients and that additional aspects such as comorbidity and frailty should be taken into account [7]. In contrast, another work showed that the chronological age of elderly patients is less important for the selection of a donor kidney than the actual physiological age. This work concluded that elderly patients should not be denied a KT solely on the basis of their age [8].

In addition to the assessment of comorbidities, sarcopenia seems to be a prognostic marker for the estimation of peri- and postoperative morbidity. Sarcopenia is the progressive and generalized deterioration of skeletal muscle [9]. In addition to the natural aging process, multiple factors play a decisive role in the development of sarcopenia in patients with terminal renal failure, including lack of exercise, chronic subliminal inflammatory processes during dialysis, metabolic acidosis, vitamin D deficiency, insulin resistance, hyperparathyroidism, and proteinuria [5, 10]. Sarcopenia correlates with outcome after gastrointestinal surgery [11, 12], as well as after liver transplantation [13]. Sarcopenia and frailty have been found to be associated with a higher risk of surgical complications [14], higher mortality [15], delayed graft function [5, 16], and a shorter graft and patient survival [5, 17]. However, the long-term effects of sarcopenia on estimates of morbidity, mortality, and organ function in KT recipients have been explored in very few studies to date, and results have been partly contradictory. A study in simultaneous pancreas-kidney transplant patients reported a non-significant trend associating low psoas muscle thickness as a parameter for sarcopenia with pancreas graft survival [18]. Druckmann et al. recently showed that the cross-sectional area of the psoas muscle is an independent factor for posttransplant mortality after KT [19]. In contrast, another study in kidney-only transplant, simultaneous liver-kidney transplant, or pancreas-kidney transplant patients showed that pre-transplant sarcopenia had no effect on posttransplant renal function or re-hospitalization rates [20].

Therefore, the aim of our study was to investigate the association between recipient age, sarcopenia, renal function, morbidity, and mortality after KT. We analyzed the impact of pre-transplant sarcopenia on graft function as the primary endpoint and its impact on secondary endpoints such as postoperative complication rates, length of hospital stay, and survival of patients after transplantation.

## Methods

This retrospective analysis was approved by the local ethics committee of the Justus-Liebig-University Giessen, Germany (institutional review board no. AZ 123/18). A total of 116 consecutive patients who had undergone KT between 2013 and 2017 were analyzed and were followed up to December 31, 2021. The collected data included the following demographic characteristics: age, sex, body mass index (BMI), underlying kidney disease, duration of dialysis, warm and cold ischemia times, intra- and postoperative course, delayed graft function, graft failure, rejection rate, surgical complications, and overall patient and graft survival data. Comorbidities were classified and analyzed by the Charlson comorbidity index [21].

Renal function was assessed using serum creatinine levels and estimated glomerular filtration rate (eGFR) according to the Chronic Kidney Disease Epidemiology Collaboration (CKD-EPI) formula at 1, 2, 3, 4, and 5 years after KT. Delayed graft function (DGF) was defined as the need for at least one dialysis within the first 7 days following KT [22].

Complications in the first 30 days after KT were classified according to the Clavien-Dindo classification [23]. The Clavien-Dindo ≥ 3a were defined as severe and included in the statistical analysis.

### Sarcopenia

Sarcopenia was defined by low skeletal muscle mass measured in computed tomography (CT) images by the sex-adjusted Hounsfield unit average calculation (HUAC). All CT scans were routinely performed during the waiting time in the last 6 months before transplantation to assess vascular status in the pelvic area.

Using syngo.via software (Siemens Healthineers, Munich, Germany), skeletal muscle mass was determined by measuring the total psoas area (TPA) at the level of the L3 vertebral body where both iliac crests were clearly visible, as described in previous publications [24, 25]. The measurements were done in a semiautomated fashion with the density threshold set between − 30 and 110 Hounsfield units (HU) to exclude vascular and fatty infiltration areas. TPA was normalized for patient’s height:

$${TPA}_{normalised}=TPA\left({mm}^2\right)\times100\;/\;body\;height\;(m^2)$$

The TPA was used to calculate the HUAC:

$$HUAC=(Right\;HUAC/TPA)+(Left\;HUAC/TPA)$$

The HUAC cut-offs to define sarcopenia were 15.69 HU in females and 16.29 HU in males [25]. The measurements were performed by a medical doctoral student (N.T.) who had previously been instructed by a radiologist in the syngo.via software and how to perform the measurements. More than one-third of the measurements were randomly selected and confirmed in a blind manner by a second investigator, an experienced radiologist (A.B.). Both investigators were blinded to the outcome parameters.

### Statistical analysis

Clinical and biochemical characteristics are expressed as the means ± standard deviations, or as medians and ranges, as appropriate. Unless otherwise indicated, all tests were two-tailed, and *p* values < 0.05 were considered significant.

Pearson’s correlation or the Spearman test was applied as appropriate to calculate the correlations between pairs of variables. Fisher’s exact test was used to analyze differences in categorical variables. For independent variables, we used the Mann–Whitney *U* and Kruskal–Wallis tests. Associations of sarcopenia with graft function were estimated using the Kaplan–Meier method, and the resulting curves were compared using the log-rank test.

To investigate risk factors for, e.g., graft failure, postoperative complications and patient survival univariate and multivariate regression analyses were performed.

Inter-rater reliability between the student’s and radiologist’s measurements was calculated to determine the extent of agreement between the individual measurements of the two examiners, along with the intra-class correlation coefficient.

All data were analyzed using SPSS, version 29 (IBM, Armonk, NY, USA).

## Results

After screening all 116 patients with KT, 5 patients were excluded because of a missing CT scan. In total, 111 kidney recipients (73 men and 38 women) were included in the present study. The patient demographics are summarized in Table 1. The mean age was 53.0 years. Among the transplants, 48.6% (*n* = 54) were living donor KTs, and 51.4% were deceased donor KT. The percentage of patients in the eurotransplant senior program (ESP) was 11.7%. A pre-emptive transplantation was performed on 12 patients (10.8%).

**Table 1:** Characteristics of kidney transplant recipients

	Total *n* = 111
Age, years (range)	53.0 (23–77)
Sex, *n* (%)	
Female	38 (34.2)
Male	73 (65.8)
BMI, kg/m^2^ (range)	25.9 (17.9–37.6)
Pre-existing conditions, *n* (%)	
Cardiovascular	83 (74.8)
Diabetes mellitus	19 (17.1)
Cerebrovascular	17 (15.3)
COPD	5 (4.5)
Charlson’s comorbidity index	
0–4	65 (58.6)
≥ 5	46 (41.4)
≥ 6	20 (18.0)
Primary renal diagnosis	
Glomerulonephritis	27 (24.3)
Polycystic kidney	19 (17.1)
Diabetes	15 (13.5)
Pyelonephritis	14 (12.6)
Hypertension	13 (11.7)
Others	17 (15.3)
Unknown	6 (5.4)
Dialysis, *n* (%)	99 (89.2)
Peritoneal dialysis	12
Hemofiltration	87
Pre-emptive transplantation, *n* (%)	12 (10.8)
Duration dialysis, years (range)	4.9 (0.2–16)
Number of patients with previous transplants, *n* (%)	12 (10.8)
Donor organ source, *n* (%)	
Deceased donor	57 (51.4)
ET senior program	13 (11.7)
Living donor	54 (48.6)
Donor characteristics	
Age, years	53.4 (16–79)
Sex, female:male, *n* (%)	62:49
BMI kg/m^2^	26.3 (19–39)
Proteinuria, *n* (%)	18 (16.2)
Creatinine, mg/dl	0.8 (0.1–4.8)
ABO incompatibility, *n* (%)	40 (36.0)
HLA mismatches > 3, *n* (%)	40 (36.0)
Length of hospital stay, days (range)	22.1 (13–71)
Cold ischemic time, minutes (range)	475.4 (66–1411)

Data are expressed as means ± standard deviations

*BMI*, body mass index; *COPD*, chronic obstructive pulmonary disease; *ET*, Eurotransplant; *HLA*, human leucocyte antigen

Most patients (74.8%) had cardiovascular diseases (hypertension, coronary heart disease, chronic cardiac failure, cardiac dysrhythmia, or valvular heart disease) in their medical history, and 17.1% had diabetes mellitus. Over 41% of the patients had the Charlson comorbidity index (CCI) ≥ 5.

The main primary renal diagnosis for ESRD was glomerulonephritis (24.3%), followed by polycystic kidney diseases (17.1%), and diabetes (13.5%).

All patients received a calcineurin inhibitor (CNI)-based immunosuppressive regimen, including tacrolimus or cyclosporine A combined with prednisolone and mycophenolate mofetil (MMF). Induction therapy was performed using basiliximab or ATG. The choice of induction therapy was based on individual patient characteristics (e.g., prior transplantation or preformed HLA-antibodies).

The outcomes after KT are shown in Table 2. Delayed graft function was seen in 13.5% of the patients and loss of graft function during the follow-up period in 15.3% patients. The main reasons for graft loss were rejections and the patient’s death. Severe complications, classified as the Clavien-Dindo ≥ 3a, were observed in 23.4%. Reasons for re-interventions were mainly surgical site infections, lymphoceles, and surgical conditions, such as thrombosis of the A. or V. renalis.

**Table 2:** Outcome after kidney transplantation

	Total *n* = 111
Primary non-function, *n* (%)	2 (1.8)
Acute rejection, *n* (%)	6 (5.4)
Delayed graft function, *n* (%)	15 (13.5)
Loss of graft function, *n* (%) Acute rejection Chronic rejection Patient’s death	17 (15.3) 4 4 9
Malignancies after Tx, *n* (%)	7 (6.3)
Surgical site infections, *n* (%)	6 (5.4)
Lymphocele, *n* (%)	10 (9.0)
Complications of the Clavien-Dindo classification ≥ 3, *n* (%)	26 (23.4)

Data are expressed as means ± standard deviations

### Sarcopenia assessment

The assessment of sarcopenia was done quickly and easily by measuring and calculating TPA and HUAC, and the results were obtained independently by two different investigators. Inter-rater reliability and the intra-class correlation coefficient were calculated with the two-way mixed model and a confidence interval (CI) of 95%. The intra-class correlation coefficient between the measurements was 0.970 (95% CI 0.942–0.984, *p* < 0.001).

The mean TPA was 519.0 mm^2^/m^2^ (94.2–914.9 mm^2^/m^2^). The mean HUAC was 17.0 HU in females (4.6–22.9 HU) and 17.9 (5.3–27.0 HU) in males. Using the previously described cut-offs for defining sarcopenia, 36 patients (32.4%) had sarcopenia.

### Impact of comorbidities and sarcopenia on graft function and outcome after kidney transplantation

Characteristics of patients stratified by sarcopenia are shown in Table 3. As expected, patients with sarcopenia were significantly older than non-sarcopenic patients (59.6 years vs. 49.8 years, *p* < 0.001). A significantly higher BMI was observed in sarcopenic patients (BMI = 27.6 kg/m^2^ vs. 25.0 kg/m^2^, *p* = 0.002). Furthermore, the possibility of sarcopenic patients receiving a deceased organ (*p* = 0.002) was significantly higher. Hospital stay was significantly longer in patients with sarcopenia compared to non-sarcopenic patients (22.7 vs. 21.8 days, *p* = 0.017). There were no significant differences between sarcopenic and non-sarcopenic patients in primary renal diagnosis, donor characteristics, cold ischemia times, immunosuppression regimen or patient survival (Suppl. Figure 1A). As expected, recipients of living donor organs were on average more than 10 years younger, had fewer pre-existing conditions, were less likely to be sarcopenic, and had a significantly shorter length of hospital stay than recipients of deceased donor organs (Suppl. Table 1). The separate analysis of the living donation and the deceased donation cohorts with regard to sarcopenia is shown in Suppl. Table 2. In both cohorts, sarcopenic patients were significantly older and had a higher BMI.

**Table 3:** Patient characteristics stratified by sarcopenia

	No sarcopenia *n* = 75	Sarcopenia *n* = 36	*p*
Sex, *n* (%)			n.s
Male	49 (65.3)	24 (66.7)	
Female	26 (34.7)	12 (33.3)	
Age, years (SD)	49.8 (12.5)	59.6 (10.6)	< 0.001
BMI, kg/m^2^ (SD)	25.0 (3.8)	27.6 (4.7)	0.002
Dialysis, *n*	63 (84.0)	36 (100.0)	0.008
Duration dialysis, years (SD)	4.1 (4.0)	4.8 (3.3)	n.s
Donor organ source, *n*			0.002
Deceased	31 (41.3)	26 (72.2)	
Living	44 (58.7)	10 (27.8)	
ET senior program, *n*	4 (5.3)	9 (25.0)	0.004
Length of hospital stay, days (SD)	21.8 (10.5)	22.7 (8.8)	0.017
TPA, mm^2^/m^2^ (SD)	539.3 (153.6)	476.6 (186.6)	n.s
HUAC, HU (SD)	19.8 (2.5)	13.0 (3.1)	< 0.001

Data are expressed as means ± standard deviations

*BMI*, body mass index; *ET*, Eurotransplant; *HUAC*, Hounsfield unit average calculation; *n.s.*, not significant; *SD*, standard deviation; *TPA*, total psoas area

We also did not observe any differences in complication rates between sarcopenic and non-sarcopenic patients. In the multivariate regression analysis, only duration of dialysis before KT was a predictor for the occurrence of complications Clavien-Dindo ≥ 3 (HR 1.157, 95% CI 1.034–1.296, *p* = 0.011). Factors such as age, gender, BMI, HUAC, and diabetes mellitus, on the other hand, were not significant.

**Fig. 1:**
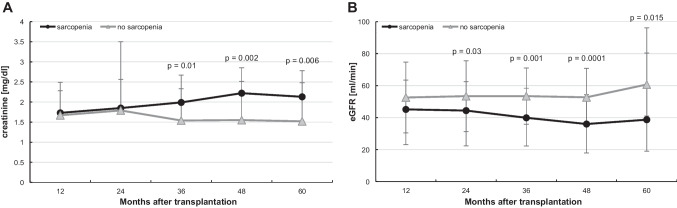
Long-term effects of sarcopenia on graft function. Kidney graft function was evaluated **A** using the serum creatinine levels and **B** on estimated glomerular filtration rates (eGFR) by the formula of the Chronic Kidney Disease Epidemiology Collaboration (CKD-EPI) at indicated time points after kidney transplantation. Patients with graft failure were excluded from this analysis. Significant *p* values are shown

Beyond that, in the first 2 years after KT, no significant difference in creatinine levels of the two groups could be observed. However, after the third year following organ transplantation, sarcopenic patients had significantly higher serum creatinine values (Fig. 1A) and significantly decreased eGRF values (Fig. 1B) than non-sarcopenic patients.

To identify factors associated with creatinine clearance in long-term (at 3 years) after KT, we performed the Pearson correlation (Table 4). There was a good correlation (*r* > 0.3) between eGFR and sarcopenia (*r* = 0.328, *p* = 0.001), transplantation in the ET senior program (*r* = 0.311, *p* = 0.002), and transplants of deceased donors (*r* = 0.406, *p* < 0.001). Donor age showed a strong negative correlation (*r* =  − 0.447, *p* < 0.001) with eGFR. Recipients’ age, Charlson’s comorbidity index, duration of dialysis, and cold ischemia time showed a significant but weak correlation with eGRF. Significant parameters were included in the multiple linear regression analysis, which identified sarcopenia as an independent variable associated with decreased eGFR at 3 years posttransplant. Deceased donor transplantation was also independently associated with decreased eGFR (*p* = 0.02) than with organs from living donors. Remarkably, sarcopenic patients also had a 9.5 times higher risk (*p* = 0.019) of decreased eGFR than non-sarcopenic patients (Table 4). For each year of donor age, eGFR decreased by 0.63 (*p* < 0.001).

**Table 4:** Analysis of transplant and donor characteristics and their association with eGFR (CKD-EPI) at the 3 years posttransplant time point

	Pearson’s correlation		Multiple linear regression analysis	
	Correlation coefficient *r*	*p*	Regression coefficient *B*	*p*
Age	− 0.263	0.009	0.033	n.s
Sex	0.035	n.s	-	
BMI	− 0.192	n.s	-	
Charlson’s comorbidity index ≥ 5	− 0.210	0.039	3.257	n.s
Dialysis	− 0.155	n.s	-	
Duration dialysis	− 0.216	0.034	− 0.183	n.s
Donor organ source	0.406	< 0.001	18.876	0.02
Donor age	− 0.447	< 0.001	− 0.630	< 0.001
Donor BMI kg/m^2^	− 0.184	n.s	-	
Donor sex	− 0.121	n.s	-	
Donor creatinine	0.051	n.s	-	
CIT	− 0.251	0.014	0.010	n.s
HLA	− 0.153	n.s	-	
ET senior program	− 0.311	0.002	2.657	n.s
Pre-transplant sarcopenia	0.328	0.001	9.478	0.019
Acute rejection	0.169	n.s	-	
DGF	− 0.099	n.s	-	
Complications	− 0.158	n.s	-	

*BMI*, body mass index; *CIT*, cold ischemia time; *COPD*, chronic obstructive pulmonary disease; *DGF*, delayed graft function; *ET*, Eurotransplant; *HLA*, human leucocyte antigen

**Fig. 2:**
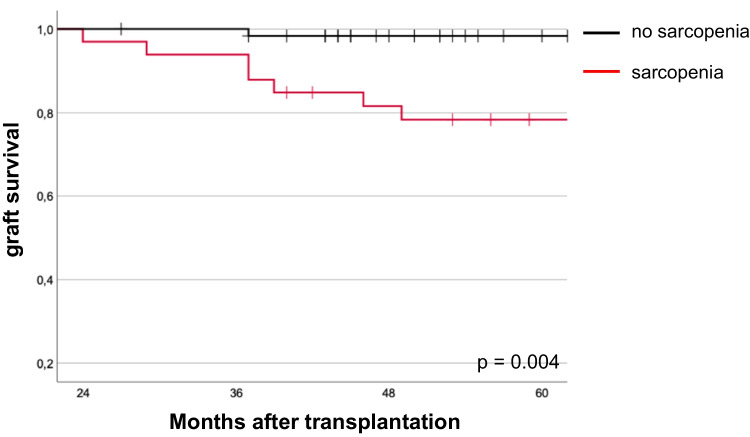
Graft survival after kidney transplantation stratified by sarcopenia. Graft survival is shown by the Kaplan–Meier analysis at indicated time points after kidney transplantation for sarcopenic and non-sarcopenic patients

The association of sarcopenia with graft survival after the second year after KT was also seen in the Kaplan–Meier analysis for graft survival. From the second year after KT onwards, the curves diverge, showing that sarcopenic patients had a significantly poorer graft survival than non-sarcopenic patients in long-term follow-up (*p* = 0.004, Fig. 2). Table 5 shows factors associated with patient and graft survival in univariate Cox’s regression analysis. For both outcome parameters, receipt of a kidney from a deceased donor, cold ischemia time, and delayed graft function are significant risk factors. Other significant factors for graft survival are also the occurrence of acute rejection with a hazard ratio of 2.6 (95% CI 1.36–4.8, *p* 0.004), but also the Charlson comorbidity index > 5 with a hazard ratio of 1.9 (1.13–3.22, *p* = 0.015). While sarcopenia is not significantly associated with patient survival, it is a risk factor for graft failure with a hazard ration of 2.6 (95% CI 1.82–5.69, *p* = 0.017). Interestingly, age is not significant here.

**Table 5:** Analysis of different transplant and donor characteristics and their association with patient and graft survival in the univariate Cox’s regression analysis

	Patient survival		Graft survival	
	Hazard ratio (95% CI)	*p*	Hazard ratio (95% CI)	*p*
Age, years	1.050 (0.999–1.104)	n.s	1.044 (0.999–1.092)	n.s
Male	1.165 (0.686–1.978)	n.s	0.903 (0.536–1.521)	n.s
BMI	1.011 (0.896–1.141)	n.s	1.010 (0.902–1.131)	n.s
Charlson’s comorbidity index ≥ 5	1.390 (0.821–2.367)	n.s	1.912 (1.134–3.221)	0.015
Dialysis	4.900 (0.125–192.028)	n.s	1.404 (0.511–3.855)	n.s
Duration dialysis	1.049 (0.930–1.183)	n.s	1.049 (0.930–1.183)	n.s
Deceased donor graft	1.923 (1.015–3.641)	0.045	1.813 (1.035–3.176	0.037
Donor age	0.992 (0.950–1.036)	n.s	1.020 (0.980–1.062)	n.s
CIT	1.001 (1.000–1.003)	0.028	1.001 (1.000–1.002)	0.037
ET senior program	1.489 (0.786–2.820)	n.s	1.266 (0.678–2.362)	n.s
Pre-transplant sarcopenia	1.427 (0.844–2.411)	n.s	2.594 (1.182–5.692)	0.017
Acute rejection	1.198 (0.433–3.318)	n.s	2.556 (1.361–4.801)	0.004
DGF	1.933 (1.152–3.447)	0.014	1.773 (1.052–2.990)	0.032
Complications	1.084 (0.339–3.464)	n.s	1.563 (0.964–2.534)	n.s

*BMI*, body mass index; *CIT*, cold ischemia time; *DGF*, delayed graft function; *ET*, Eurotransplant; *n.s.*, not significant

Nine patients, who all received a deceased donor graft, died during the follow-up time (range 16–42 months after KT) with a functioning graft. Five patients were sarcopenic before KT. Detailed patient characteristics are shown in Table 6.

**Table 6:** Characteristics of deceased patients, *n* = 9

ID	Age at KT, years	Sarcopenia	Graft failure	Survival (month)	Cause of death
17	47	No	No	37	Cardiovascular diseases
22	59	No	No	19	Cancer
26	67	Yes	No	10	Infection/sepsis
28	70	Yes	No	40	Infection/sepsis
35	68	No	No	37	Cardiovascular diseases
36	69	Yes	No	13	Infection/sepsis
55	62	Yes	No	23	Cancer
72	66	Yes	No	19	Infection/sepsis
103	62	No	No	39	Cancer

*KT*, kidney transplantation

## Discussion

The increasing number of patients with ESRD is challenging in transplant settings, especially as sarcopenia and frailty were found to be associated with a higher risk of surgical complications [14], higher mortality [15, 19], delayed graft function [5, 16], and shorter graft and patient survival [5, 17]. In this study, we analyzed the impact of pre-transplant sarcopenia on graft function, postoperative complication rates, and survival. Firstly, the proportion of patients with sarcopenia in our study was high—32.4%. Previously, other studies have shown sarcopenia rates between 20 and 25% [26, 27]. However, these results were obtained in KT recipients who were on average over 10 years younger than our patients. In recipients of simultaneous liver-kidney transplantation, rates as high as 72% have been reported [28].

Secondly, we confirmed that sarcopenia was associated with reduced graft function and graft survival from the third year after KT. Our work showed a strong association of sarcopenia with serum creatinine levels, eGFR, and graft survival in long-term after KT. Streja et al. used low serum creatinine as a surrogate for low muscle mass in a large registry data analysis and concluded that low muscle mass tends to result in poorer graft survival [29]. Interestingly, in a study of a patient collective that had received simultaneous pancreas-kidney transplantation, significantly lower pancreas survival, but not kidney survival, was seen in sarcopenic patients [30]. In our work, we showed that, in addition to sarcopenia, donor age and use of organs from deceased donors were associated with poorer transplant function in the long term. 

Thirdly, sarcopenic patients had a significantly longer hospital stay after KT. Our findings are supported by the work of Druckmann et al., who showed that pre-transplant sarcopenia is not only associated with increased postoperative morbidity but also with longer hospital stay [19]. In another recent study, a higher re-admission rate was observed in sarcopenic patients after a deceased organ transplant in the first 30 days after the KT [31]. The association between sarcopenia and length of hospital stay has been also reported in patients who have received liver transplants [32, 33]. The long length of hospital stay in our cohort is explained by the high rate of ABO-incompatible transplants (36%) and the associated special treatment protocols. The number of ABO-incompatible transplant patients in our study did not differ significantly between the sarcopenic and non-sarcopenic cohorts, nor between KT recipients after living donation or after deceased kidney donation.

However, the significance of the KT recipient’s sarcopenia before KT for the outcome after KT remains unclear, and the data are partly contradictory. A recently published retrospective, single-center study in 573 KT recipients (including simultaneous liver-kidney and pancreas-kidney transplants) found no significant impact of pre-transplant sarcopenia on eGFR, graft loss, posttransplant mortality, and hospitalization rates [20].

In addition to age-related processes, sarcopenia in potential KT recipients is also worsened by various factors, including malnutrition, metabolic acidosis, accumulation of uremic toxins, and amino acid loss during dialysis, as well as typical low-threshold inflammation [34]. Malnutrition is very common in patients requiring dialysis and leads to sarcopenia in these patients by reducing muscle mass and strength as well as physical performance [35]. Furthermore, dialysis patients have a lower physical activity compared to healthy people of the same age, which in turn increases muscle wasting [36]. Malnutrition and lack of physical activity can be important targets for the prevention of sarcopenia and its associated morbidity and mortality in patients with ESRD.

While there are only a few publications dealing with sarcopenia as a predictor of kidney graft outcome [5, 16, 19, 20, 30, 31], a large number of studies published in the last years show an increased postoperative morbidity and mortality after visceral surgery and in oncologic patients [37–43]. A meta-analysis explored independent risk factors for postoperative complications and patient’s death in patients with resected lung cancer [44]. A study by Ishida et al. observed a significantly worse response rate to neoadjuvant chemotherapy, higher rate of postoperative complications, and an unfavorable survival rate in sarcopenic patients with curative intended esophageal cancer [45]. Interestingly, low skeletal muscle areas combined with high visceral fat were associated with a worse outcome in colon cancer patients, together with an increased expression of proinflammatory and inhibition of anti-inflammatory cytokines [46]. 

However, despite the high number of publications showing sarcopenia to be a predictive marker of outcome, the methods used to determine sarcopenia varied widely: questionnaire (SARC-F), hand grip strength, muscle mass measured using bioelectrical impedance, dual-energy X-ray absorptiometry, or CT or magnetic resonance imaging (MRI) [47, 48]. A preferred method when using CT or MRI scans is to estimate skeletal muscle mass and density [49]. The psoas muscle is very well suited to diagnosing sarcopenia, as it does not change in chronic illnesses but not in acute illnesses but in chronic ones [19, 49]. We showed that the assessment of sarcopenia with the measurement and calculation of TPA and HUAC of the psoas muscle was simple, fast, easy, and valid when performed by non-radiology employees. In particular, since most KT recipients receive a CT scan during their waiting time, for example to assess the vascular status in the pelvic area, these patients therefore do not require an additional CT examination to assess sarcopenia.

With this simple but effective method for the classification of sarcopenic and non-sarcopenic patients, an optimized selection of recipients can be made, and the appropriate patient cohort for pre-habilitation can already be identified during the waiting period. Sarcopenia can be avoided or improved by exercise therapy and nutritional measures.

This study has limitations due to the retrospective nature of the data collection. Not all possible risk factors for kidney recipients were available, especially environmental, behavioral, and psychological factors. Due to the low number of patients, multivariate analyses for risk factors for, e.g., graft failure and loss of graft function, were not possible. Nevertheless, we observed a distinct negative effect on graft function in the sarcopenic subgroup.

## Conclusions

Sarcopenia in KT recipients was very common and was a negative predictor of long-term graft function and graft survival. Furthermore, sarcopenic patients have a significantly longer stay in hospital after transplantation. The method we have presented for assessing sarcopenia using CT scans is quick, easy, and suitable for non-radiology professionals.

Early identification of sarcopenic patients is important as it would allow pre-habilitation programs to be initiated while patients are on the waiting list for a donor organ. In addition to reducing sarcopenia, patients at risk of developing sarcopenia could also be identified and treated in an interdisciplinary manner, including nutritional and exercise therapy. Therapy or avoidance of sarcopenia could not only improve the outcome after KT, but also reduce the length of hospital stay. However, prospective randomized studies are required for this.

The authors declare that the results presented in this paper have not been published previously in whole or part, except in abstract format.

### Supplementary Information

Below is the link to the electronic supplementary material.

Supplementary file1 (PPTX 1117 KB)

Supplementary file2 (DOCX 24 KB)
