# Supplementary material for: Sarcopenia of kidney transplant recipients as a predictive marker for reduced graft function and graft survival after kidney transplantation
Source: Langenbecks Arch Surg. 2023 Feb 24;408(1):103. doi: 10.1007/s00423-023-02836-1 (PMC9958183; doi:10.1007/s00423-023-02836-1)
Supplement: Supplementary file 1 — Supplementary file1 (PPTX 1117 KB) [file 423_2023_2836_MOESM1_ESM.pptx]

## Slide 1
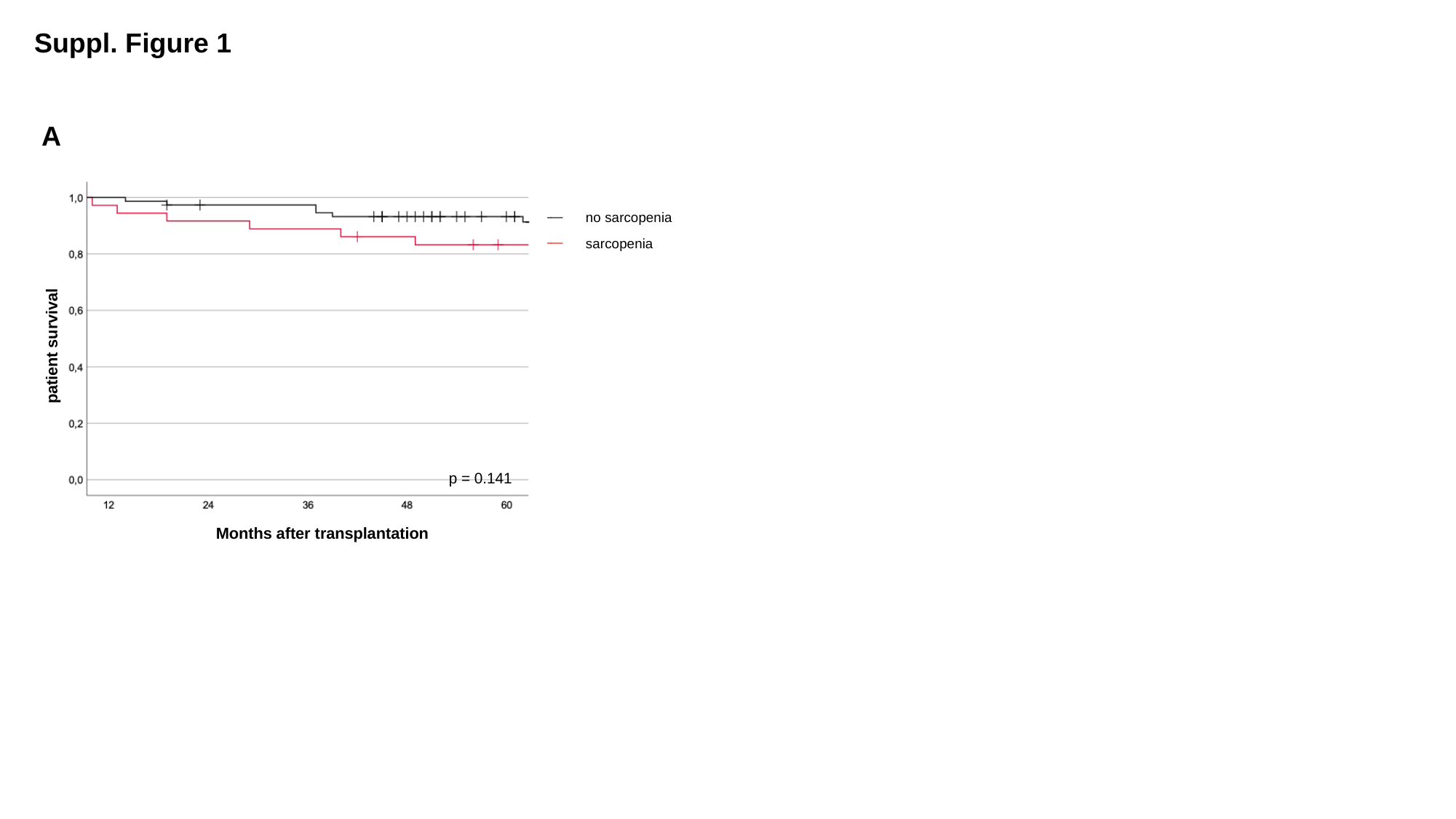

Suppl. Figure 1
A
__
__
no sarcopenia
sarcopenia
patient survival
p = 0.141
Months after transplantation
