# Supplementary material for: Sarcopenia of kidney transplant recipients as a predictive marker for reduced graft function and graft survival after kidney transplantation
Source: Langenbecks Arch Surg. 2023 Feb 24;408(1):103. doi: 10.1007/s00423-023-02836-1 (PMC9958183; doi:10.1007/s00423-023-02836-1)
Supplement: Supplementary file 2 — Supplementary file2 (DOCX 24 KB) [file 423_2023_2836_MOESM2_ESM.docx]

**Supplementary Table 1: Characteristics stratified by donor organ source**

|  | **Living donation**  **n = 54** | **Deceased donation**  **n = 57** | **p** |
| --- | --- | --- | --- |
| Age, years (SD) | 47.6 (13.2) | 58.1 (10.0) | **< 0.001** |
| Gender, n (%)   - Female - Male | 19 (35.2)  35 (64.8) | 19 (33.3)  38 (66.7) | n.s. |
| BMI, kg/m^2^ (SD) | 25.3 (3.9) | 26.4 (4.5) | n.s. |
| Pre-existing diseases, n (%)   - Cardiovascular - Diabetes mellitus - Cerebrovascular - COPD | 33 (61.1)  8 (14.8)  3 (5.6)  3 (5.6) | 50 (87.7)  11 (19.3)  14 (24.6)  2 (3.5) | **0.002**  n.s.  **0.007**  n.s. |
| Charlson Comorbidity Index   - 0 – 4 - ≥ 5 - ≥ 6 | 39 (72.2)  11 (20.4)  4 (7.4) | 5 (8.8)  35 (61.4)  17 (29.8) | **< 0.001** |
| Dialysis, n (%)   - Peritoneal dialysis - Hemofiltration | 43 (79.6)  6 (11.1)  37 (68.5) | 56 (98.2)  6 (10.5)  50 (87.7) | **0.003** |
| Pre-emptive transplantation, n (%) | 11 (20.4) | 1 (1.8) | **0.002** |
| Duration dialysis, years (SD) | 2.2 (2.9) | 6.3 (3.5) | **< 0.001** |
| Number of patients with previous transplants, n (%) | 6 (11.1) | 6 (10.7) | n.s. |
| Donor characteristics   - Age, years (SD) - Gender, female:male, n - BMI kg/m^2^ (SD) - Proteinuria, n (%) - Creatinine, mg/dl | 51.4 (9.9)  32:22  26.4 (4.1)  0  0.79 (0.27) | 55.4 (14.5)  30:27  26.2 (3.7)  18 (31.6)  0.8 (0.7) | n.s.  n.s.  n.s.  **< 0.001**  n.s. |
| ABO incompatibility, n (%) | 23 (42.6) | 17 (29.8) | n.s. |
| HLA mismatches > 3, n (%) | 19 (35.2) | 21(36.8) | n.s. |
| Length of hospital stay, days (SD) | 19.5 (6.9) | 24.5 (11.7) | **0.007** |
| Cold ischemic time, minutes (SD) | 108.9 (26.1) | 835.3 (284.7) | **< 0.001** |
| TPA, mm^2^/m^2^ (SD) | 537.6 (164.7) | 501.3 (168.3) | n.s. |
| HUAC, HU (SD) | 19.1 (3.6) | 16.2 (4.2) | **< 0.001** |
| Pretransplant sarcopenia, n (%) | 10 (18.5) | 26 (45.6) | **0.003** |
| Primary non-function, n (%) | 0 | 2 (3.5) | n.s. |
| Acute rejection, n (%) | 2 (3.7) | 4 (7.0) | n.s. |
| Delayed graft function, n (%) | 1 (1.9) | 14 (24.6) | **< 0.001** |
| Surgical site infections, n (%) | 1 (1.9) | 5 (8.8) | n.s. |
| Lymphocele, n (%) | 5 (9.3) | 5 (8.8) | n.s. |
| Complications Clavien-Dindo ≥ 3, n (%) | 10 (18.5) | 16 (28.1) | **0.002** |

Data are expressed as means ± standard deviations. BMI = body mass index; COPD = chronic obstructive pulmonary disease; HLA = human leucocyte antigen; HUAC = Hounsfield Units Average Calculation; TPA = total psoas area.

**Supplementary Table 2: Characteristics stratified by donor organ source and sarcopenia**

|  | **Living kidney donor recipients** | | | **Deceased kidney donor recipients** | | |
| --- | --- | --- | --- | --- | --- | --- |
|  | No Sarcopenia  n=44 | Sarcopenia  n=10 | p | No Sarcopenia  n=31 | Sarcopenia  n=26 | p |
| Sex, n (%)   - Male - Female | 28 (63.6)  16 (36.4) | 7 (70)  3 (30) | n.s. | 21 (67.7<9  10 (32.3) | 17 (65.4)  9 (34.6) | n.s. |
| Age, years (SD) | 46.7 (12.9) | 51.7 (14.8) | n.s. | 54.2 (10.6) | 62.7 (6.7) | **< 0.001** |
| BMI, kg/m^2^ (SD) | 24.8 (3.3) | 27.1 (5.8) | **0.05** | 25.2 (4.4) | 27.8 (4.3) | **0.016** |
| Charlson Comorbidity Index ≥ 5 | 9 (20.5) | 2 (20) | n.s. | 16 (51.6) | 19 (73.1) | n.s. |
| Dialysis, n | 33 (75.0) | 10 (100) | n.s. | 30 (96.8) | 26 (100) | n.s. |
| Duration dialysis, years (SD) | 2.1 (3.0) | 2.8 (2.1) | n.s. | 7.0 (3.5) | 5.6 (3.4) | n.s. |
| Donor characteristics   - Age, years (SD) - Sex, male:female, n - BMI, kg/m^2^ (SD) | 51.1 (9.7)  18:26  26.4 (4.1) | 52.9 (11.4)  4:6  26.3 (4.2) | n.s.  n.s.  n.s. | 54.7 (13.7)  15:16  26.2 (3.7) | 56.2 (15.5)  12:14  26.2 (3.8) | n.s.  n.s.  n.s. |
| ET Senior Program, n | 0 (0) | 0 (0) | n.s. | 4 (12.9) | 9 (34.6) | **0.001** |
| HLA mismatches > 3, n (%) | 15 (34.1) | 4 (40) | n.s. | 11 (35.5) | 10 (28.5) | n.s. |
| ABO incompatibility, n (%) | 16 (36.4) | 7 (70) | n.s. | 11 (35.5) | 6 (23.1) | n.s. |
| Length of hospital stay, days (SD) | 19.2 (6.8) | 20.9 (7.2) | n.s. | 25.5 (13.4) | 23.4 (9.4) | n.s. |
| TPA, mm^2^/m^2^ (SD) | 539.1 (164.6) | 531.0 (173.7) | n.s. | 539.6 (139.1) | 455.6 (190.4) | **0.03** |
| HUAC, HU (SD) | 20.3 (2.6) | 13.9 (2.5) | **< 0.001** | 19.1 (2.2) | 12.7 (3.2) | **< 0.001** |

Data are expressed as means ± standard deviations. BMI = body mass index; ET = Eurotransplant; HLA = human leucocyte antigen; HUAC = Hounsfield Units Average Calculation; n.s. = not significant; SD = standard deviation; TPA = total psoas area
